# Biomass-Derived Carbon Utilization for Electrochemical Energy Enhancement in Lithium-Ion Batteries

**DOI:** 10.3390/nano14120999

**Published:** 2024-06-08

**Authors:** Byeong Jin Jeong, Feng Jiang, Jae Yoon Sung, Soon Phil Jung, Dae Won Oh, RM. Gnanamuthu, Kumaran Vediappan, Chang Woo Lee

**Affiliations:** 1 Department of Chemical Engineering (Integrated Engineering Program), College of Engineering, Kyung Hee University, 1732 Deogyeong-Daero, Giheung, Yongin 17104, Gyeonggi, Republic of Korea; byjin98@khu.ac.kr (B.J.J.); bong0201@gmail.com (F.J.); jyoons1994@khu.ac.kr (J.Y.S.); sprichwortj@khu.ac.kr (S.P.J.); od9942@khu.ac.kr (D.W.O.); 2 Center for the SMART Energy Platform, College of Engineering, Kyung Hee University, 1732 Deogyeong-Daero, Giheung, Yongin 17104, Gyeonggi, Republic of Korea; 3 Centre for Nonlinear System, Chennai Institute of Technology, Chennai 600 069, Tamil Nadu, India; gnanamuthu.chem@gmail.com; 4 Department of Chemistry, Faculty of Engineering and Technology, SRM Institute of Science and Technology, Kattankulathur 603 203, Tamil Nadu, India

**Keywords:** potato peel, biomass, lithium-ion batteries, lithium iron phosphate, nitrogen-doped carbon

## Abstract

Cathodes made of LiFePO_4_ (LFP) offer numerous benefits including being non-toxic, eco-friendly, and affordable. The distinctive olivine structure of LFP cathodes contributes to their electrochemical stability. Nonetheless, this structure is also the cause of their low ionic and electronic conductivity. To enhance these limitations, an uncomplicated approach has been effectively employed. A straightforward solid-state synthesis technique is used to apply a coating of biomass from potato peels to the LFP cathode, boosting its electrochemical capabilities. Potato peels contain pyridinic and pyrrolic nitrogen, which are conducive to ionic and electronic movement and facilitate pathways for lithium-ion and electron transfer, thus elevating electrochemical performance. When coated with nitrogen-doped carbon derived from potato peel biomass (PPNC@LFP), the LFP cathode demonstrates an improved discharge capacity of 150.39 mAh g^−1^ at a 0.1 C-rate and 112.83 mAh g^−1^ at a 1.0 C-rate, in contrast to the uncoated LFP which shows capacities of 141.34 mAh g^−1^ and 97.72 mAh g^−1^ at the same rates, respectively.

## 1. Introduction

Recently, with their advantages of high energy and power density, lithium-ion batteries (LIBs) have been enormously consumed in mobile devices, electric vehicles, and energy storage devices [1,2,3,4]. At present, cathode active materials that have been widely studied typically include layered structures (Li[Ni_x_Co_y_Mn_1−x−y_]O_2_, Li[Ni_0.8_Co_0.15_Al_0.05_]O_2_), spinel structures (LiMn_2_O_4_), and olivine structures (LiFePO_4_) [2,5,6,7,8]. In particular, olivine-structured LiFePO4 (LFP) has lots of merits such as high thermal stability, low material cost, and non-toxicity, and it has become one of the first choices for electric power devices [9]. However, the electronic and ionic conductivities of LFP are limited by its structure. This is attributed to the P-O bond, a strong covalent bond, which restricts the movement of the Li-ion. Hence, it makes the practical capacity of LFP unable to reach its theoretical capacity and limits the rate capability and high-rate performance [9,10,11]. To solve these problems, many researchers have conducted compositional and surficial modification studies, such as doping and coating [12,13,14]. Carbon coating techniques might be the most effective strategy to elevate the electrochemical performance of LFP. The conductivity of LFP is extremely poor, so the high conductivity of carbon can compensate for one of its defects [15,16,17,18]. Moreover, it can prevent direct contact between electrolytes and electrodes, preventing an unwanted attack within the reaction [19]. In addition to carbon coatings, heteroatom doping in carbon is also widely used because heteroatoms, such as B, S, F, and N, can be doped into carbon and improve its electrochemical characteristics [20,21]. Doping heteroatoms into carbon structures can make defects and lithium ions (Li-ions) easier to embed and help move them to the cathode [21,22,23]. Among heteroatoms, the N atom is the most widely used, mainly because it is easy to extract.

Biomass, such as nutshells, peels, algae, and shellfish, has been highlighted as a carbon source and it can reduce environmental pollution and convert it into energy [24]. The N atom, which exists in some biomass, can reduce additional doping process, resulting in lessening the synthesis cost [25,26,27]. Potato is widely known as one of the world’s food crops. The world produces 376 million tons of potatoes yearly, which also leads to the possibility of generating massive potato waste [28]. So, if discarded potato peel is utilized for energy sources [29,30,31], it can help recycle biomass, which boosts environmental sustainability. According to Osman et al., carbonized potato peel contains two nitrogen–doped carbon components, pyridinic nitrogen and pyrrolic nitrogen, which positively affect LIBs [22,32,33,34,35].

In this study, using inexpensive and abundant potato peel biomass, a simple and effective solid-state synthesis method is used to prepare nitrogen-doped carbon coating for LFP and its effect on the physico-chemical and electrochemical properties is diversely investigated.

## 2. Materials and Methods

### 2.1. Synthesis Method of Nitrogen-Doped Carbon-Coated LFP

The potatoes were first peeled and then the potato peels were washed with distilled water and dried for approximately one day. Dried potato peels were then evenly mixed. Thereafter, carbonization process was adopted at 300 °C for 3 h under a N_2_ gas. A certain amount of carbonized potato peels and 3 g of LFP (STL) were thoroughly mixed and thermally treated under a N_2_ gas at 600 °C for 3 h. The resulting sample was denoted as potato peel biomass-derived nitrogen-doped carbon-coated LFP (PPNC@LFP). 

### 2.2. Electrochemical Measurements

The materials used in electrode synthesis are LFP active materials, Denka Black and Polyvinylidene fluoride (PVdF), and they were ground at a ratio of 85:10:5 with an appropriate amount of N-methyl-2-pyrrolidone (NMP) for a slurry. The slurry was laminated on an aluminum foil and dried overnight at room temperature. The cathode was then dried in a convection oven for 5 h at 120 °C. Then, the cathode was pressed and punched into a disk with a 14 mm diameter as a cathode in coin cells. Finally, the punched cathode was placed in a vacuum oven for 5 h at 120 °C. The electrochemical performance of the prepared cathode was assessed using a 2032-type coin cell. The anode was a lithium foil, the electrolyte was 1 M LiPF_6_ salt dissolved in ethylene carbonate (EC) and diethyl carbonate (DEC) (1:1 vol.%), and Celgard^®^2320 (Charlotte, NC, USA) was used for the membrane separator. The coin cells were assembled in an Ar gas-filled glove box and aged for 12 h. The electrochemical performance of the prepared cells was assessed using a battery cycler (ETH, Suwon, South Korea) and electrochemical impedance spectroscopy (EIS) analyses of LFP were carried out using an electrochemical workstation (Iviumstat, Ivium Technologies, Eindhoven, The Netherlands).

### 2.3. Material Characterizations

The crystal structure and phase purity of the as-prepared materials were studied using X-ray diffraction (XRD, Rigaku Miniflex 600, Tokyo, Japan) in the 2θ range between 10° and 60°. Raman spectroscopy was used to analyze the functional properties using a Raman Confocal Microscope (HR-Raman, Renishaw, Wotton-under-Edge, Gloucestershire, UK). X-ray photoelectron spectroscopy (XPS, Multilab 2000, Thermo VG Scientific, Waltham, MA, USA) was used to analyze the elemental composition of the materials. Morphological and elemental analysis and elemental mapping were performed using a high-resolution field-emission scanning electron microscopy (HR FE-SEM, MERLIN-LEO SUPRA 55, Carl Zeiss, Oberkochen, Germany) and a field-emission transmission electron microscopy (FE-TEM, JEM-2100 F; JEOL, Tokyo, Japan) equipped with energy-dispersive spectroscopy (EDS), respectively.

## 3. Results

### 3.1. Physical and Chemical Characterizations

**Figure 1:**
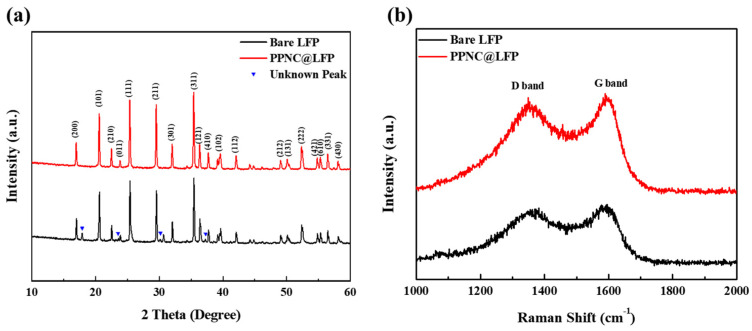
(**a**) XRD patterns of bare LFP and PPNC@LFP; (**b**) Raman spectra of bare LFP and PPNC@LFP.

The XRD patterns of bare LFP and PPNC@LFP are shown in Figure 1a. LFP (JCPDS 40-1499) [14] has an orthonormal homomorphic structure and belongs to the *Pnma* space group [11]. Bare LFP shows unknown peaks as observed in the XRD pattern analysis. We acquired bare LFP from an external chemical supplier and these peaks are likely due to undefined additives that do not negatively affect the electrochemical performance [34]. During the synthesis of nitrogen-doped carbon-coated LFP, the unidentified peaks completely disappeared after high-temperature calcination, and no further impurity peaks were observed. The lattice parameters of LFP and PPNC@LFP are summarized in Table 1. The results did not show any significant differences in the crystal structure, and the carbon coating layer also did not affect the lattice parameter and the unit cell volume of LFP.

Figure 1b shows the carbon microstructure analysis performed using Raman spectroscopy. As shown in the figure, the peaks correspond to the D and G bands of bare LFP and PPNC@LFP, with values of 1360 cm^−1^ and 1600 cm^−1^, respectively. The D and G bands, which exhibit the disorder and graphite properties of carbon, are associated with the bending and elongation modes of all sp^2^ atoms in the carbon ring [23]. The degree of graphitization was expressed as the ratio of the intensity (*I_D_*/*I_G_*) of the D and G bands, and the intensities of bare LFP and PPNC@LFP are *I_D_*/*I_G_* = 0.8404 and *I_D_*/*I_G_* = 0.8512, respectively. The intensity of graphite property of bare LFP is greater than that of PPNC@LFP; that is, the carbon disorder property of PPNC@LFP is well exhibited, indicating a higher conductivity than that of bare LFP [21,36].

**Figure 2:**
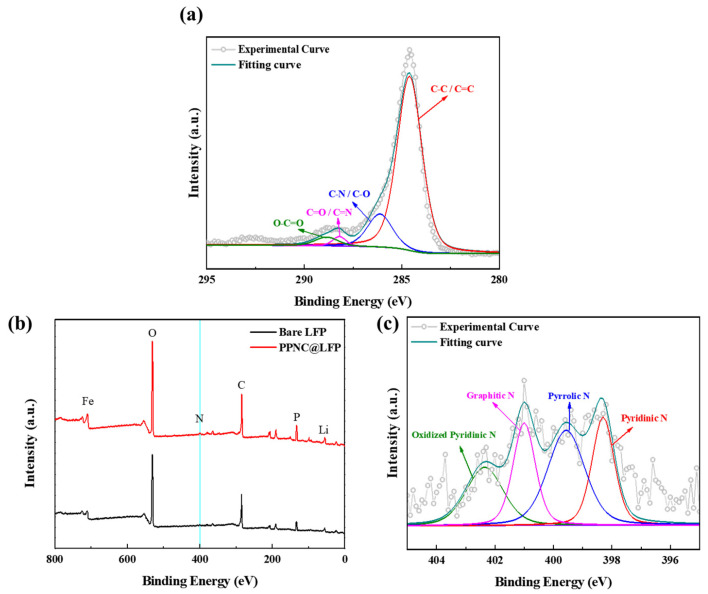
(**a**) XPS survey profiles of bare LFP and PPNC@LFP; (**b**) XPS multiple profiles and fitting curves of C 1s for PPNC@LFP; (**c**) XPS multiple profiles and fitting curves of N 1s for PPNC@LFP.

Figure 2a shows the XPS spectra of bare LFP and PPNC@LFP, found in the nitrogen appearance only in PPNC@LFP, confirming that a nitrogen-doped carbon coating was formed from potato peels. Figure 2b displays the XPS spectrum of C 1s for PPNC@LFP. The carbon is confirmed as a C−C/C=C bond, C−O/C−N bond, C=O/C=N bond, and O=C−O bond and their values of binding energy are 284.6 eV, 286.1 eV, 288.1 eV, and 288.8 eV, respectively [23,37,38]. Furthermore, in Figure 2c, the multiple profiles of the N 1s orbital of PPNC@LFP are confirmed to be pyridinic nitrogen (23%), pyrrolic nitrogen (34%), graphitic nitrogen (22%), and oxidized pyridinic nitrogen (21%) and their values of binding energy are 398.2 eV, 399.5 eV, 400.9 eV, and 402.3 eV, respectively [28,39,40]. The emergence of pyridinic nitrogen and pyrrolic nitrogen are attributed to the thermally induced decomposition of protein, which is one of the ingredients of potato peels. Pyridinic nitrogen and pyrrolic nitrogen provide active sites for the movement of Li-ions and electrons, promoting ionic and electronic conductivities [19,33,41].

**Figure 3:**
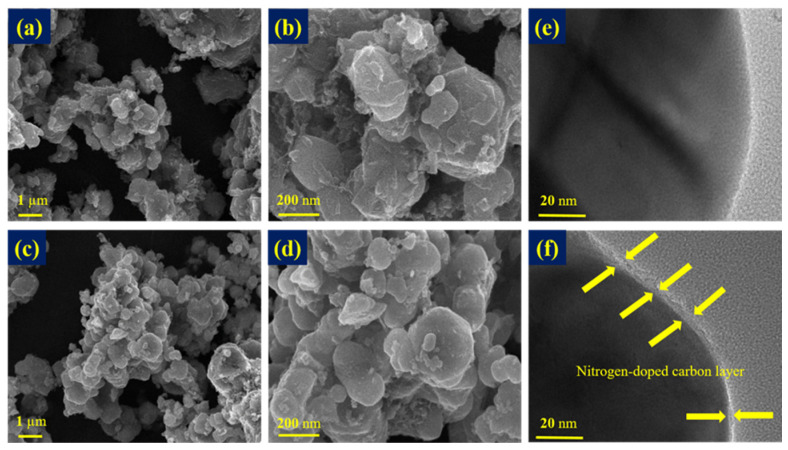
HR FE-SEM images of (**a**,**b**) bare LFP and (**c**,**d**) PPNC@LFP; FE-TEM images of (**e**) bare LFP and (**f**) PPNC@LFP.

**Figure 4:**
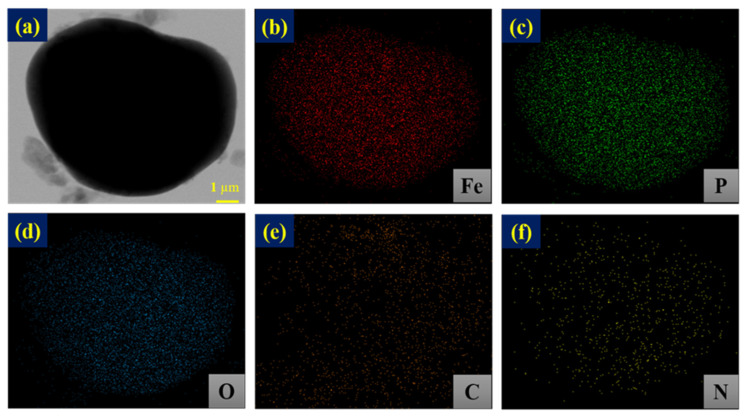
FE-TEM images of (**a**) PPNC@LFP and EDS spectra of (**b**–**f**) EDS mapping images of Fe, P, O, C, and N, respectively.

Figure 3a–d display the HR FE-SEM images of bare LFP and PPNC@LFP and reveals that there is a limit for PPNC@LFP to identify the layer formation on the surface of LFP after coating. Figure 3e,f present images characterized via FE-TEM, and the image analysis shows that the nitrogen-doped carbon was coated on the LFP surface. The coating layer thickness is approximately 4 nm, and an appropriate amount of the coating layer is advantageous for Li-ions to move, preventing an unwanted attack from by-products caused by the reaction between the electrode and the electrolyte [19]. Figure 4 shows the EDS mapping analysis, indicating that Fe, P, O, C, and N were distributed on the surface of LFP and explaining the existence of a nitrogen-doped carbon coating on the surface of LFP.

### 3.2. Electrochemical Performances

**Figure 5:**
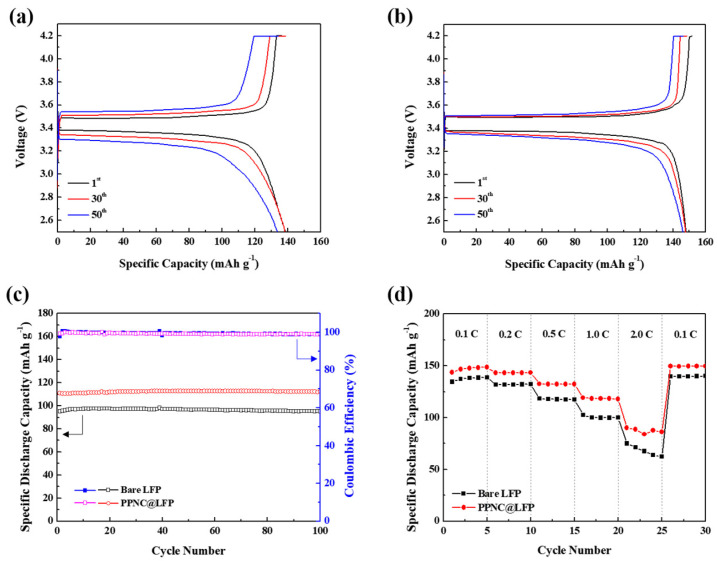
Galvanostatic charge–discharge potential profiles for (**a**,**b**) bare LFP and PPNC@LFP; (**c**) cycling performance profiles showing specific discharge capacity between the voltage window of 2.5–4.2 V at a current rate of 1.0 C and its coulombic efficiency of bare LFP and PPNC@LFP; (**d**) rate capability.

The galvanostatic charge–discharge shown in Figure 5 compares the electrochemical performance of bare LFP and PPNC@LFP at the range of 2.5~4.2 V. 

Based on the experiments performed at 0.1 C for 50 cycles, Figure 5a,b show the graphs for the specific discharge capacity of bare LFP and PPNC@LFP with values of 141.34 mAh g^−1^ and 150.39 mAh g^−1^, respectively. Figure 5c shows the cycling performance of bare LFP and PPNC@LFP at 1.0 C for 100 cycles and their coulombic efficiency. The initial specific discharge capacities of bare LFP and PPNC@LFP were 97.72 mAh g^−1^ and 112.15 mAh g^−1^, respectively. After 100 cycles, the specific discharge capacities were 95.22 mAh g^−1^ and 112.08 mAh g^−1^. The capacity retention of bare LFP was calculated to be lower than that of PPNC@LFP. Figure 5d presents the rate capability of bare LFP and PPNC@LFP on the various current density ranges and PPNC@LFP exhibits the highest specific discharge capacity. The specific discharge capacity gap between the attempted materials was more significant at 2.0 C and both materials show a good recovery in the specific discharge capacity when it returned to 0.1 C. It was confirmed that the electrochemical performance was improved via nitrogen-doped carbon coating, demonstrating that nitrogen-doped carbon coatings can effectively prevent direct contact between the electrode and the electrolyte and inhibit excessive metal dissolution. Additionally, N-substitution of carbon resulted in defects in the carbon ring structure, improving the electron and ion transfer, as shown in the Raman spectra and XPS. This indicates that the electronic and ionic conductivities of PPNC@LFP were higher than those of bare LFP. Therefore, nitrogen-doped carbon coating from potato peels can effectively improve the cyclic stability and discharge capacity.

**Figure 6:**
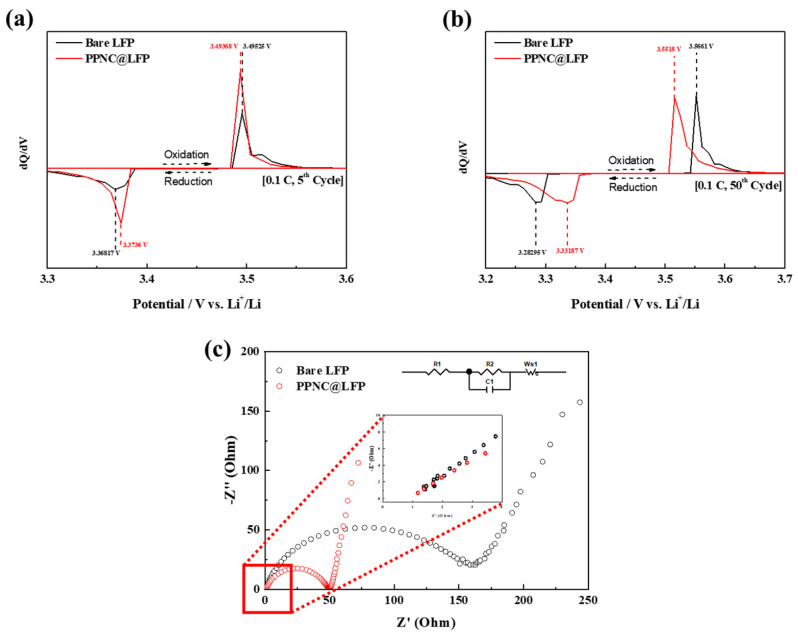
(**a**,**b**) Differential capacity curves; (**c**) EIS analysis after fully charged at 1.0 C of bare LFP and PPNC@LFP.

Figure 6a,b show the differential capacity curves (dQ/dV) with the potential change of the 5th and 50th cycles at 0.1 C for bare LFP and PPNC@LFP. In the charge–discharge cycle, oxidation and reduction in Fe-ion peaks are redox processes in LFP [34]. In Figure 6a, for the fifth cycle, the oxidation peaks of bare LFP and PPNC@LFP were 3.49525 V and 3.49368 V, and the reduction peaks were 3.36817 V and 3.3736 V. The potential differences (ΔE) of the oxidation and reduction peaks were 0.12433 V and 0.12008 V, respectively. For the 50th cycle, as shown in Figure 6b, the oxidation peaks of bare LFP and PPNC@LFP were 3.5518 V and 3.56614 V, while the reduction peaks were 3.28295 V and 3.33187 V and their potential differences (ΔE) were 0.26885 V and 0.22427 V, respectively. Polarization is related to Li-ion conductivity, so PPNC@LFP is better than bare LFP on both the 5th and 50th cycles, indicating that PPNC@LFP has better ionic conductivity. Also, the electrochemical performance and the ionic conductivity of biomass-derived carbon-coated LFP cathode materials are compared in Table 2. Among the biomass-derived carbons, LFP coated by the orange peel presents the lowest ionic conductivity of 4.80 × 10^−4^ S cm^−1^. The eggshell membrane had similar electrochemical performance to that of the potato peel but was measured to be relatively less conductive than the potato peel. As shown in Table 2, PPNC@LFP exhibits the highest ionic conductivity of 2.52 × 10^−3^ S cm^−1^.

Figure 6c shows the EIS analysis using 2032-type coin cells made of bare LFP and PPNC@LFP. Preceded by full charging, the cell was investigated after 100 cycles at 1.0 C in the frequency range of 100 mHz to 10 kHz. The charge transfer resistance (*R_ct_*) conforms to the semicircle in the Nyquist plots in the high-frequency region [34]. The *R_s_* and *R_ct_* values of PPNC@LFP are 1.05 Ω and 49.94 Ω, respectively, whereas those of bare LFP are 1.37 Ω and 158.27 Ω. The *R_ct_* value of PPNC@LFP was lower than that of bare LFP, indicating that the nitrogen-doped carbon coating improved the electrochemical reaction between the electrode and the electrolyte [19]. Additionally, PPNC@LFP had a smaller semicircle which demonstrates the tendency of the charge to decrease, reducing the charge transfer resistance [44]. This suggests that the coating layer protects the bulk region of LFP without obstructing the Li-ion and electron pathways. This indicates that the synergistic effect of the nitrogen-doped carbon coating increases the charge transfer interface reaction efficiencies and carbon randomness and improves the conversion of nitrogen-doped carbon into LFP [21]. On the coating side, resistive reactions that occur between the electrode and the electrolyte are suppressed, preferably preventing direct contact with each other. 

## 4. Conclusions

Nitrogen-doped carbon from potato-peel-coated LFP was obtained through a simple solid-state synthesis method. The specific discharge capacities of PPNC@LFP at 0.1 C and 1.0 C are 150.39 mAh g^−1^ and 112.83 mAh g^−1^, respectively, indicating a 10% capacity improvement when compared with those of bare LFP. Furthermore, PPNC@LFP demonstrates about 99.9% of cyclic stability for 100 cycles at 1.0 C. The crystal structure and morphology of LFP are not significantly changed, and the resistance between the electrode and the electrolyte is reduced after using nitrogen-doped carbon. In PPNC@LFP, we can observe pyridinic nitrogen and pyrrolic nitrogen which provide Li-ion and electron channels, enhancing conductivity. Therefore, the attempted biomass-type potato peel would be an attractive alternative effectively able to enhance the electrochemical performance of LFP. 

## Figures and Tables

**Table 1:** Lattice parameters of LFP and PPNC@LFP.

Sample	a (Å)	b (Å)	c (Å)	V (Å^3^)
Bare LFP	10.336	6.007	4.694	291.451
PPNC@LFP	10.316	6.001	4.688	290.242

**Table 2:** Comparison of the biomass-derived carbon-coated LFP cathode materials.

Carbon Precursor	Cathode Active Material	Discharge Capacity (mAh g^−1^)	Cycling Performance	Ionic Conductivity (S cm^−1^)	Ref.
Orange peel	LFP	147.3 at 0.5 C	95% after 50 cycles at 0.5 C	4.80 × 10^−4^	[42]
Eggshell membrane	LFP	155.4 at 0.1 C	99% after 100 cycles at 1.0 C	1.04 × 10^−3^	[35]
Dopamine	LFP	163 at 0.1 C	97.9% after 50 cycles at 0.1 C	-	[43]
Potato peel	LFP	150 at 0.1 C	99% after 100 cycles at 1.0 C	2.52 × 10^−3^	This work

## Data Availability

Data are contained within the article.
